# Toward a porcine *in vivo* model to analyze the pathogenesis of TLR5-dependent enteropathies

**DOI:** 10.1080/19490976.2020.1782163

**Published:** 2020-07-25

**Authors:** Robert Pieper, Niels van Best, Kira van Vorst, Friederike Ebner, Monika Reissmann, Mathias W. Hornef, Marcus Fulde

**Affiliations:** a Institute of Animal Nutrition, Freie Universität Berlin, Berlin, Germany; b Institute of Medical Microbiology, RWTH University Hospital Aachen, Aachen, Germany; c Institute of Microbiology and Epizootics, Freie Universität Berlin, Berlin, Germany; d Institute of Immunology, Freie Universität Berlin, Berlin, Germany; e Humboldt-Universität zu Berlin, Berlin, Germany

**Keywords:** Toll-like receptor 5, metabolic syndrome, inflammatory bowel disease, microbiota, pig model

## Abstract

Non-communicable diseases, such as the metabolic syndrome and inflammatory bowel disease, constitute serious public health threats in developed countries. Besides environmental factors, genetic predispositions contribute to the onset and progression of the disease. *State-of-the-art* mouse models recently highlight the involvement of Toll-like receptor 5 (TLR5)–driven microbiota composition in the development of metabolic disorders. Here, we discuss the causes and consequences of an altered enteric microbiota and provide information on a similar mechanism in another species, the pig. We show for the first time that a single nucleotide polymorphism in the porcine *TLR5* gene conferring impaired functionality is associated with changes in the intestinal microbiota in adult sows and neonatal piglets. Changes in the developing adaptive cellular immune response support the concept of TLR5-driven changes of the microbe-host interplay also in the pig. Together, these findings suggest that pigs with impaired TLR-functionality might represent a model for TLR5-driven diseases in humans.

## Introduction

The propagation of vaccination by Edward Jenner in 1796 and the discovery of penicillin by Sir Alexander Fleming in 1928 dramatically changed the prospects of medicine. At this time, infectious diseases, such as tuberculosis, diphtheria, typhoid fever and smallpox represented the major causes for human mortality. In contrast, non-communicable diseases such as cancer, cardiovascular and metabolic diseases are among the primary causes of mortality in industrialized countries today. The underlying pathologies remain incompletely understood but both genetic predisposition and environmental factors were shown to contribute to the etiology and disease progression.^1,2^ As a consequence, current preventive measures and interventional strategies target mechanisms of disease progression and clinical symptoms.

Major advances in the DNA-sequencing technology in the past years led to the renaissance of the so-called “forgotten organ,” the enteric microbiota. The intestinal microbiota includes all microorganisms, bacteria, viruses, fungi and parasites that colonize the mucosal surfaces of the gastrointestinal tract of warm- and cold-blooded animals.^3^ It is known that the microbiota significantly supports the physiology of its host providing essential vitamins, degrading otherwise indigestible nutritional constituents enhancing the energy harvest and stimulating and educating the innate and adaptive mucosal and systemic immune system.^4^ On the other hand, alterations in the microbiota composition have been associated with a wide spectrum of metabolic, inflammatory and immune-mediated disorders and appear to also contribute to the etiology or progression of at least some highly prevalent diseases.^5,6^

Metabolic syndrome (MetS) and inflammatory bowel disease (IBD) constitute two disease entities associated with an altered microbiota composition.^7–9^ For example, a reduced bacterial diversity, an increased number of mucolytic bacterial species and/or so-called pathobionts (e.g. adherent-invasive *Escherichia coli* (AIEC)) were associated with IBD.^10^ Similarly, also the microbiota of patients suffering from MetS constitute a lower bacterial richness compared to non-obese individuals.^11^ Recent data suggest that the observed compositional changes of the microbiota may play a functional role in the disease pathogenesis.^12–15^ In addition, also genetic factors were identified to contribute significantly.^16–18^ The clinical symptoms of IBD, MetS and type-2 diabetes mellitus are reminiscent of the phenotype of mice deficient for the innate immune receptor Toll-like receptor 5 (TLR5), the receptor for bacterial flagellin. TLR5-deficient mice develop hyperphagia, obesity, insulin resistance, glucose tolerance, hypertension, hyperlipidemia and an inflamed intestinal mucosa.^19,20^ Interestingly, in several American, Indian and Danish cohorts, the development of IBD was found to be significantly associated with the occurrence of single nucleotide polymorphisms (SNPs) in the human *TLR5* gene.^21,22^ Also, microbiota analyses revealed significant differences in the composition and diversity of the enteric microbiota between TLR5-proficient and TLR5-deficient mice.^20,23^ Two experimental approaches suggested that the microbiota of TLR5-deficient mice was causally related to the development of metabolic symptoms. First, antibiotic treatment corrected the metabolic phenotype in TLR5-deficient mice. Second, the transfer of the microbiota from adipose TLR5-deficient mice to healthy germfree wildtype animals led to the development of metabolic symptoms in the recipient animals.^19^ Notably, the question, why healthy, TLR5-proficient germfree animals failed to counteract the development of metabolic symptoms by correcting the dysbiotic microbiota remained unanswered until recently. We and others reported that the expression of TLR5 by intestinal epithelial cells (with the exception of Paneth cells) is strictly age-dependent with high expression restricted to the pre-weaning period.^23,24^ Consistently, neonate but not adult mice were able to suppress intestinal colonization by flagellated bacteria in a TLR5-dependent manner by the induction of a mucosal antimicrobial response. Subsequently, comparative transfer of the microbiota from adult TLR5-proficient and deficient donor animals revealed that only neonate TLR5-proficient mice but not adult TLR5-proficient or TLR5-deficient mice were able to correct for the TLR5-dependent difference. Thus, genetic factors contribute to the composition of the enteric microbiota. In mice, the influence might be age-restricted, i.e. act only during an early “window of opportunity.” Once established, the stability of the enteric microbiota then might have life-long consequences and determines the susceptibility to metabolic and inflammatory symptoms in the adult host. Notably, our results thereby highlight the critical and non-redundant role of the postnatal period for the life-long host-microbial and immune homeostasis.

Interestingly, variants in the TLR5 locus were also found in other animals, e.g. in the German shepherd dog. Notably, also here an association between inherited TLR5 dysfunction and protection from IBD and other enteropathies was established.^25,26^ Furthermore, the pig breed “Deutsche Landrasse” harbors an SNP in *TLR5*, which is inherited recessively on chromosome 10 and leads to a P402L amino acid change causing impaired recognition of *Salmonella* and flagellin, respectively.^27^ This SNP does not lead to an entirely dysfunctional TLR5 but has been shown to reduce downstream NF-κB activation by about one-third as compared to the wildtype TLR5 allele.^28^ Consistently, the impaired flagellin recognition in the pig may confer an increased susceptibility to flagellated bacteria, such as *Salmonella* Typhimurium and (entero-)pathogenic *E. coli*.^29–31^ The latter observation opens the possibility for further investigations of the functional role of TLR5-signaling in mucosal inflammation in a model that mimics the human situation more closely and may additionally allow the evaluation of potential preventive measures and therapeutic strategies. Pigs share key similarities with humans, such as body size, anatomical features, (patho-)physiological responses and diet.^32^ Also, the microbiota of humans is more similar to the microbiota of pigs than rodent animals.^33^ This is particularly important since microbiota alterations appear to play a functional role.^12^

**Figure 1.**
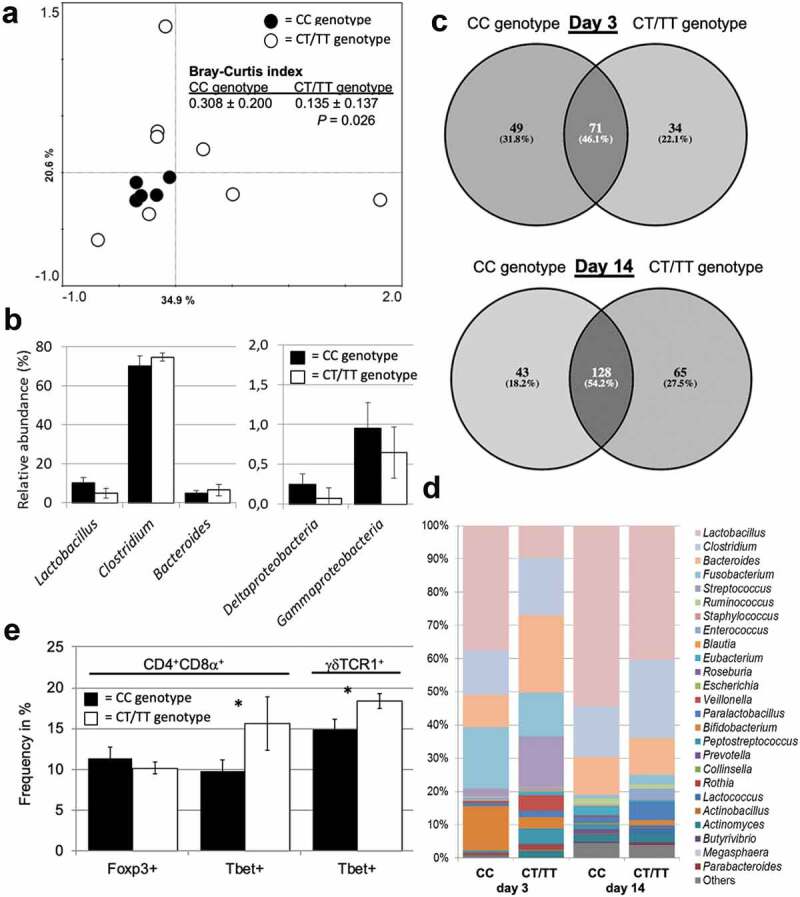
The SNP c.1205C>T in the porcine *TLR5* gene is associated with alterations in the enteric microbiota in adult and neonatal pigs as well as the developing adaptive immune response. (a) PCA plot of fecal microbial communities of adult sows with CC genotype (n = 5) or CT/TT genotype (n = 8). Bray-Curtis index was used to determine the intra-group similarity of microbial communities. (b) Relative abundance of major microbial groups in adult sows. (c) Venn diagrams illustrating the number of unique and shared bacterial taxa in fecal samples of neonatal piglets at days 3 or 14 of age with CC genotype (n = 3) or the CT/TT genotype (n = 3). (d) Relative abundance of major microbial groups in fecal samples of neonatal piglets at 3 or 14 d after birth. (e) Phenotypic characterization of T cell polarization of neonatal piglets at 14 d after birth by flow cytometric analysis of peripheral blood immune cells. The C allele represents the “wild type” allele (functional TLR5), whereas the T allele represents the “mutant” allele with impaired flagellin recognition.

In preliminary experiments, we have established a rapid screening assay for the above-mentioned SNP conferring impaired functionality of TLR5 (C1205T) in pigs and retrospectively screened a set of adult animals (n = 13 sows) and their offsprings at days 3 and 14 after birth (n = 6, respectively). During the gestational period, the sows were group-housed and moved into individual pens 10 d prior to farrowing, where they remained with their piglets during the suckling period. Principal component analysis of the fecal microbiome composition of sows during the gestational period revealed different patterns between individuals carrying the CC wildtype genotype as compared to individuals carrying the CT or the TT genotype (Figure 1(a)). As further shown by significant differences in the Bray–Curtis index, sows carrying the CC genotype exhibited a more similar fecal microbiota (reduced within-group distance) as compared to sows with CT or TT genotype. Western-type diet and gestational age have previously been linked to markers for metabolic syndrome in pig models.^34,35^ Since the sows investigated here were co-housed, bred in the same environment and fed the same diet during the study period, the genotype might play a significant role in the gut microbiota composition. The data suggest that the fecal microbiota of sows with the CC genotype are characterized by a higher abundance of lactobacilli and certain *Proteobacteria*, whereas pigs with the CT/TT genotype exhibit a higher abundance of *Clostridium* and *Bacteroides* (Figure 1(b)). Whether these differences are comparable to microbiota shifts described in humans suffering from MetS or IBD or the corresponding rodent models will require further investigations.^23,36,37^ It remains to be clarified whether the differences in the enteric microbiota of adult sows are a consequence of long-term selection by TLR5 during the establishment of the intestinal microbiota between birth to adulthood.^38^ As a first indication for such an early-life selection process, the fecal microbiota of neonatal piglets grouped according to the *TLR5* genotype showed a high degree of unique bacterial taxa (53.9% at day 3, 45.8% at day 14 of life). Notably, these differences were observed despite the fact that piglets were kept in the same environment and that some of them were even siblings (Figure 1(c)). Concomitantly, differences in the abundance of certain high-abundant genera at day 3 (e.g. *Lactobacillus, Fusobacterium, Streptococcus*,) and day 14 (e.g. *Lactobacillus, Clostridium, Ruminococcus*) after birth point toward a TLR5-driven colonization pattern also in the neonate piglet (Figure 1(d)). These patterns could have long-lasting effects on gut microbiota composition until adulthood, the immune system development and host metabolism. These data support our previously published results in the rodent model^23^ and suggests that the pig with impaired TLR5-functionality might indeed represent a promising model for TLR5-driven enteropathies. A preliminary analysis of peripheral blood mononuclear T cell subsets was performed in the neonatal piglets to evaluate a putative influence on the adaptive immune system (Figure 1(e)). A significant difference in type-1 polarization of differentiated T effector cell subsets of both TCR-αβ^+^ (CD3^+^CD4^+^CD8α^+^) and TCR-γδ^+^ (CD3^+^CD4^−^CD8α^+^γδTCR1^+^) T lymphocytes was detected. T cells expressing the transcription factor Tbet were higher abundant in neonatal piglets with the CT/TT genotype as compared to the “wild type” CC genotype. Interestingly, at this age, no differences were observed for the abundance of T regulatory cells (i.e. CD3^+^CD4^+^CD8α^+^ T cells expressing the transcription factor Foxp3). Together, consistent with the mouse model, these data show for the first time that impaired pattern recognition by TLR5 leads to a disturbed microbiome and a dysregulated immune cell composition in pigs. Both observations support a putative role in the pathogenesis of chronic enteropathies (i.e. IBD and MetS). Thus, as recently proposed by the Caplice group that established a porcine model of diet- and mineralocorticoid-induced MetS,^39^ the pig might represent an interesting model to study the link between an altered gut microbiota, its influence on the immune system and potential consequences on the mucosal host-microbial homeostasis in more detail. Since TLR5 impairment or deficiency is an established genetic risk factor for the development of MetS or IBD, the porcine animal model might open new avenues for the biomedical research of non-communicable diseases associated with TLR5. Studies with this model should also include cross-fostering studies and dietary interventions to further unravel the effect of the genotype and distinguish it from environmental and dietary effects in the etiology of MetS and IBD.^40^

### Supplemental Methods related to Figure 1

All procedures involving pig handling and treatments were approved by the local state office of occupational health and technical safety (LAGeSo Reg. #0269/16). Multiparous sows (n = 13) were kept in a commercial barn at the Institute of Animal Nutrition (Freie Universität Berlin, Berlin, Germany) and fed commercial gestation diets without any medical intervention during the last two parities. Approximately 1 month prior to farrowing a fecal and blood sample was collected from each sow for genotyping. A total of n = 12 piglets from six sows were also genotyped as described below. The piglets were kept with their mothers in individual pens. Fecal samples were taken from n = 6 neonatal piglets at day 3 and 14 after birth, respectively, for microbiota analysis. On day 14 of life, blood samples were taken from all genotyped piglets for phenotypic characterization of peripheral blood mononuclear cells.

### Fecal microbiome analysis

Total genomic DNA from fecal samples was extracted using a commercial kit (Qiagen Stool kit, Qiagen, Hilden, Germany) according to the manufacturer’s instructions except for an increase in temperature during the lysis step to 90°C. Sample preparation, including amplification of the V3 region of the 16 S rRNA gene using primer set 341 F-785 R, equimolar mixing, sample clean-up and sequencing by Illumina MiSeq, was performed by LGC Genomics (Berlin, Germany). In addition, a subset of fecal samples (adults) has been isolated by Repeated-Bead-Beating (RBB) combined with chemical lysis plus a column-based purification method as described in detail elsewhere,^41^ and sequencing of the V3-V4 region was performed according to previously published protocols.^41,42^ Pooled amplicons were purified using AMPure XP purification (Agencourt, Massachusetts, USA) and subsequently quantified by Quant-iT PicoGreen dsDNA reagent kit (Invitrogen, New York, USA). Amplicons were mixed in equimolar concentrations and sequenced on an Illumina MiSeq instrument. Data demultiplexing, length and quality filtering, pairing of reads and clustering of reads into Operational Taxonomic Units (OTUs) at 97% sequence identity were done using the online Integrated Microbial Next Generation Sequencing (IMNGS) platform^43^ using default settings except for minimum and maximum length for amplicons, which were set at 300 and 600 bp, respectively. Demultiplexed and primer-clipped sequence data of all other samples were uploaded to the MG-RAST Server (https://www.mg-rast.org/) and processed by its SEED software tool. The phylogenetic profile of each sample was computed with the following parameters: maximum e-value of 1e-6, minimum percent identity of 96% and minimum alignment length of 150 bases. The Green Gene reference data bank was used for identification. Bacterial taxa with five or less identical sequence reads per sample were removed from further analysis. Similarly, sequence reads occurring in one sample only were ignored. Remaining sequence reads were used to calculate the relative contribution of specifically assigned sequences to total sequence reads in a sample.

### *TLR5* genotyping

The SNP c.1205C>T in the porcine TLR5 gene was genotyped with an allele-specific PCR method using StepOnePlus Real-Time PCR System (Applied Biosystem).^44,45^ The primers (A1: CAAGAAGAGAGTAGGTATGCTCG; A2: CCAAGAAGAGAGTAGGTATGCTCA; C: CCGGGATAATGCTCTTAAAACAATTCAGTT) were designed and produced as ready to use primer assay mix by Biosearch Technologies. The PCR reaction was carried out in a volume of 8.0 µl containing 30 ng dried DNA, 4.0 µl reaction mix (Biosearch Technologies), 0.11 µl primer assay mix, 0.06 µl 50 mM Mg^2+^ and 3.83 µl water under the following conditions: 94°C for 15 min followed by 10 touch down cycles with 94°C for 20 sec and touch down of 0.8°C per cycle from 65°C to 57°C for 60 sec. Additional, 26 cycles with 94°C for 20 sec and 57°C for 60 sec were carried out. Finally, the allele-specific fluorescent intensity was measured.

### Immune cell phenotyping

Mononuclear cells from porcine peripheral blood were isolated by density centrifugation of diluted (1:2 in 0.9% NaCl) blood using Pancoll solution (density 1.077 g/ml, PAN-Biotech, Aidenbach, Germany) as described previously.^46^ Cells were stained with the following antibodies specific for pig species: anti-TCR1δ-unlab (clone PGBL22A, isotype IgG1, Kingfisher Biotech, Saint Paul, MN, USA), anti-CD3ε-PerCP-Cy5.5 (clone BB23-8E6-8C8, BD Biosciences, Heidelberg, Germany), anti-CD4α-Pe-Cy7 (clone 74–12-4, BD Biosciences, Heidelberg, Germany), anti-CD8α-AlexaFluor® 647 (clone 76-2-11, BD Biosciences, Heidelberg, Germany). The following cross-reactive primary or secondary antibodies were used: anti-Foxp3-Pb (clone FJK-16s, eBioscience), anti-T-bet-PE (clone 4B10, BioLegend®, San Diego, CA, USA) and anti-mouse IgG1-FITC (clone M1-14D12, eBioscience, Thermo Fisher, Waltham, USA). Fixable viability dye was used in eFluor® 780 (eBioscience, Thermo Fisher, Waltham, USA). Intracellular transcription factors were stained after fixation and permeabilization of cells (Transcription Factor Staining Buffer Set, eBioscience, Thermo Fisher, Waltham, USA). Cells were acquired using a MACS Quant flow cytometer (Miltenyi, Bergisch-Gladbach, Germany), and post-acquisition data analysis was carried out using MACSQuantify software (Miltenyi Biotech GmbH, Bergisch-Gladbach, Germany).

Lymphocytes were identified based on their forward and side scatter properties followed by doublets and dead cell exclusion using a viability dye. Porcine, double-positive TCR-αβ Th cells were categorized based on the markers CD3^+^CD4^+^CD8α^+^ whereas TCR-γδ T cells were classified as CD3^+^CD4^−^γδTCR1^+^.

### Statistical analysis

Principal component analysis (PCA) of sow microbiome data was performed with CANOCO statistical package using the relative abundance data of individual OTUs.^47^ Intra-group similarity of microbial communities was determined using Bray–Curtis index for animals having the CC genotype or CT/TT genotype, respectively. Comparison of Bray–Curtis index, relative abundance of microbial groups as well as T cell subsets between *TLR5* genotypes was done by t-test in SPSS (version 25, Chicago, IL, USA). Venn diagrams illustrating unique or shared individual bacterial taxa in fecal microbiomes of neonatal piglets with different *TLR5* genotypes were created using Venny 2.1 (https://bioinfogp.cnb.csic.es/tools/venny/).
